# A Case of Resection of the Elbow-Joint

**Published:** 1842-09-17

**Authors:** Thomas Harris

**Affiliations:** Surgeon U. S. N.


### MEDICAL EXAMINER.

NEW SERIES.

No. 38.] PHILADELPHIA, SEPTEMBER 17, 1842. [Vol. I.

### *A Case of Resection of the Elbow-Joint.*

By Thomas Harris, M. D., Surgeon U. S. N. Reported with Remarks by Meredith Clymer, M. D.

Mary P——t, æt. 26, native of Ireland, married, and has resided in the United States five years. Eighteen months previous to the operation she fell on her left elbow. At the time she suffered but little pain, but four weeks subsequently she experienced a good deal of suffering, and the limb was so much bruised as to prevent her working. This soon passed off, and she apparently recovered from the effects of the injury. About six months afterwards she was kicked by a cow on the wrist of the same arm, and was a good deal hurt. The wrist became swollen and very painful, and the swelling extended to the elbow-joint, which became so stiff as to prevent motion. She was admitted into the Surgical wards of the Pennsylvania Hospital, August 6th, 1834. On examination of the arm, a fracture at the elbow-joint was discovered. Union occurred after a lapse of several months, but was followed by inflammation, which terminated in abscess and extensive caries. She was treated in the usual manner, a free communication being kept up externally, without success, until the 1st June, 1835, when it was decided that an operation was demanded.

On the 6th of June, 1834, the operation of resection of the elbow-joint was performed in the amphitheatre of the Pennsylvania Hospital. The method employed was that of Moreau, and since revived and extensively employed by Mr. Symes of Edinburgh. The limb was semi-flexed. A longitudinal incision was made on the side of the internal condyle, commencing about two inches above its tuberosity, and terminated at the articulation; a similar one was made on the other side, and the two united by a transverse cut, which latter divided the triceps immediately above the olecranon process. This quadrilateral flap was detached from below upwards. The muscular structure was then separated from the joint, and the condyles of the humerus, with its articulating surface, and that of the radius, and the olecranon process of the ulna, were removed by the bone nippers.

During the operation the patient suffered a great deal of pain, which continued for some hours, and the subsequent depression was very alarming. There was impeded respiration with difficulty of articulation and deglutition, which were relieved by sinapisms, stimulating enemata, &c.

June 7th. Patient seems better. Pulse 84; skin cool and moist; tongue clean; slept well; little appetite; no thirst.

June 8th. Complains of a good deal of pain in the joint on the slightest motion. Considerable cough; (this existed for some weeks prior to the operation;) slight fever; no appetite. Cold applications to the head, and cold drinks.

June 9th. Wound dressed this morning; looks very healthy; free discharge of good pus; at several points union by the first intention has occurred; slept well; no headache; considerable pain in the joint; bowels not opened since yesterday; appetite not improved. Injection of molasses, salt and water, to be followed by a laxative powder; diet, arrow root and sago.

June 10th. Bowels freely moved, and the patient feels comfortable, with the exception of the pain in the joint, which does not diminish. Pulse good; appetite better; no thirst. The wound is dressed daily with simple dressing, and presents a very favourable aspect.

June 12th. The patient improves, and suffers less pain during the dressing. She sleeps well; tongue clean and moist; bowels open.

June 14th. Not as well as at the last report. A little febrile excitement, with slight gastritis, and an increase of pain in the joint. Thirst constant; skin hot and dry. Neutral mixture and cooling drinks ordered. Wound dressed this morning; continues to discharge freely pus of a good character; adhesive strips all removed, and union by the first intention has occurred in many places.

10 o'clock, P. M. Secondary hæmorrhage, preceded by great pain, which awakened the patient, took place to the extent of f℥xvj. It was arrested by compression, and the patient feels every way comfortable; pulse not sensibly affected by the loss of blood.

June 16th. No return of hæmorrhage; the patient is still feeble, but the wound presents the same character. A small quantity of Madeira wine ordered in the food she takes. Bowels opened by a common enema.

June 18th. No change worthy of noting since the last report. A general improvement is manifested.

From this time she daily grew better. She occasionally suffered violent pain in the stomach and bowels, but the condition of the joint steadily improved.

[The above case is published from notes taken at the time. Seven years have now elapsed, and when last seen, a year since, the woman was doing very well. The wound had not reopened; considerable motion was preserved, enough, indeed, to enable the patient to use her hand for all ordinary purposes. It is proper to state that for some months prior to the operation, and at the time it was performed, the woman was labouring under severe constitutional symptoms and hectic fever. These disappeared in the progress of the cure. This is the first time the operation was performed in this country. In January, 1841, Dr. Gurdon Buck, one of the surgeons of the New York Hospital, operated for the second time. The final result of Dr. Buck's operation was not made known, to our knowledge. It has recently been performed by our friend Dr. Pancoast, the Professor of Anatomy in the Jefferson Medical College, and in our next number we hope to present our readers with the case in detail. We lately mentioned a case (p. 564) which came under our notice in Paris a year since, where fourteen years subsequent to the operation motion was nearly perfect. Messrs. Mannoury and Thore, late internes of Mr. Roux in the Hôtel Dieu, mention a case seen two years after the operation, where "motion was entirely rëestablished:" and Mr. Robert exhibited on the 5th of last June, to the Academy of Medicine at Paris, a woman in whom he had excised the elbow-joint two years and a quarter previously, and where there was recovery of motion, (p. 589.) Since August, 1840, Mr. Roux has replaced the incision in H for the one in T, a modification which he thinks facilitates the after treatment; prevents motion of the limb in the dressing, and hastens subsequent reunion. Mr. Liston adopts also this method. Since 1812, Mr. Roux has performed this operation 18 times, and with 5 deaths only.] M·C.

